# Identification and characterization analysis of sulfotransferases (*SOTs*) gene family in cotton (*Gossypium*) and its involvement in fiber development

**DOI:** 10.1186/s12870-019-2190-3

**Published:** 2019-12-30

**Authors:** Liyuan Wang, Xiyan Liu, Xiaoyang Wang, Zhaoe Pan, Xiaoli Geng, Baojun Chen, Baoshen Liu, Xiongming Du, Xianliang Song

**Affiliations:** 1 State Key Laboratory of Crop Biology/Agronomy College, Shandong Agricultural University, Taian, 271018 China; 2 State Key Laboratory of Cotton Biology, Institute of Cotton Research, Chinese Academy of Agricultural Sciences, Anyang, 455000 China

**Keywords:** Sulfotransferases (*SOTs*), Cotton, Phylogenetic analysis, Expression and regulation, Fiber development

## Abstract

****Background**:**

Sulfotransferases (*SOTs*) (EC 2.8.2.-) play a crucial role in the sulphate conjugation reaction involved in plant growth, vigor, stress resistance and pathogen infection. *SOTs* in Arabidopsis have been carried out and divided into 8 groups. However, the systematic analysis and functional information of *SOT* family genes in cotton have rarely been reported.

****Results**:**

According to the results of BLASTP and HMMER, we isolated 46, 46, 76 and 77 *SOT* genes in the genome *G. arboreum*, *G. raimondii*, *G. barbadense* and *G. hirsutum*, respectively. A total of 170 in 245 *SOTs* were further classified into four groups based on the orthologous relationships comparing with *Arabidopsis*, and tandem replication primarily contributed to the expansion of *SOT* gene family in *G. hirsutum*. Expression profiles of the *GhSOT* showed that most genes exhibited a high level of expression in the stem, leaf, and the initial stage of fiber development. The localization analysis indicated that *GhSOT67* expressed in cytoplasm and located in stem and leaf tissue. Additionally, the expression of *GhSOT67* were induced and the length of stem and leaf hairs were shortened after gene silencing mediated by *Agrobacterium*, compared with the blank and negative control plants.

****Conclusions**:**

Our findings indicated that *SOT* genes might be associated with fiber development in cotton and provided valuable information for further studies of *SOT* genes in *Gossypium*.

## Background

Sulfur is one of the most basic elements in the plant life. Its assimilation in higher plants and the decrease of metabolically important sulfur compounds are key factors in plant growth, vigor and stress resistance [1]. Sulfur plays an important role in the structure, regulation and catalysis of proteins. According to the previous study, sulfation is essential for nodulation factors of rhizobia to signal to plants in bacteria [2]. In mammals, sulfation contributes to the homeostasis and regulation of many endogenous compounds with biological activity [3]. In plants, the sulphate conjugation reaction appears to play an important part in plant growth, development and stress adaptation [4]. Sulfate must be activated by two subsequent activation steps to form adenosine-5′-phosphosulfate (APS) and 3′-phosphoadenosine-5′-phosphosulfate (PAPS) before being used for biochemical conversion [5].

Sulfotransferases (*SOTs*) (EC 2.8.2.-) catalyze the transfer of a sulfate group from PAPS to a hydroxyl group of different substrates [6]. The first plant *SOT* gene was cloned from *Flaveria* species (*Asteraceae*), which was related to the sulfation reaction of flavonol [7]. Subsequently, the cDNA encoding sulfotransferase was isolated from *Arabidopsis thaliana* and its deduced 302 amino acid polypeptide was highly correlated with plant flavonol sulfotransferase [8]. *SOTs* are widespread among higher plants, animals and eubacteria [1, 9]. Based on previous studies, *SOT* proteins were involved in the regulation of diverse physiological and biological processes, such as growth, development, adaptation to land, stomatal closure, drought tolerance and pathogen infection [1, 3, 8–18]. *SOTs* of *Flaveria* species were well characterized by means of molecular biology and biochemistry and used as a general model of plant *SOTs* [7]. These *SOTs* accept different flavonols as sulfate receptors, which may be involved in adaptation to stress or polar auxin transport. When *Arabidopsis* seedlings were treated with hormones or stress-related compounds, *SOT* protein expression was significantly induced by salicylic acid and methyl jasmonate. In addition, the accumulation of *SOTs* was also observed in the leaves or cell suspensions of mature plants after infection with bacterial pathogens [8]. Several other reports revealed that *SOTs* can directly catalyze thioglucosate, brassinosteroid, jasmonate, flavonoids and salicylic acid, and directly or indirectly participated in defense signaling, development and stress responding [1, 10, 12, 16, 19].

Cotton (*Gossypium*) is a major industrial crop that provides important natural fibers and edible oil in the world. The genus contains 45 diploid and 5 tetraploid species. Among them, *Gossypium hirsutum* L. has been cultivated worldwide and currently accounts for the vast majority of the world’s fiber output (> 90%) of the world’s fiber production [20–22]. The cotton fiber is a unique elongated cell, which is helpful to study cell differentiation. Cotton fibers are single-cell trichomes differentiated that has undergone four major developmental stages, including initiation, elongation, secondary cell wall synthesis, and maturity [23]. The development of cotton fibers in elongation and secondary cell wall synthesis determines the length and strength characteristics of the fiber [24]. In addition, fiber development is a complex process involved in many pathways, including various secondary metabolism, hormone, signal transduction and transcriptional regulatory components [25, 26]. For example, one of the flavonoids, naringenin has been verified to be negatively correlated with fiber development [26, 27]. Auxin and brassinosteroid promoted the fiber initiation as well as elongation; gibberellin acid and ethylene played a positive role during the fiber elongation phase [25–29]. On the other hand, cytokinin, abscisic acid played an opposite role [11]. Jasmonic acid participates in various developmental processes. Different concentrations of jasmonic acid play different roles and high concentration of jasmonic acid inhibits fiber initiation [30, 31]. Similarly, jasmonate inhibited cotton development to some extent by inhibiting gibberellin signal [32]. Overaccumulation of jasmonic acid inhibited both lint and fuzz fiber initiation, reduced the fiber length, and lead to a fiberless phenotype in cotton seeds [33].

Considering that *SOTs* directly catalyze brassinosteroid, jasmonate, flavonoids and salicylic acid, which are related to growth, cotton fiber development and stress adaptation, it is necessary to understand the information of *SOT* gene family in *Gossypium* in order to better understand the relationship between sulfation reaction and physiological processes. However, as far as we know, there is no systematic study of the *SOT* family in *Gossypium*. In this study, we identified 46, 46, 76 and 77 *SOT* genes from *G. arboreum*, *G. raimondii*, *G. barbadense*, and *G. hirsutum*, respectively, and then looked into the features such as chromosomal locations, phylogenetic evolutionary relationships, gene structures, conserved motifs, tissue and subcellular localization, as well as expression patterns. Our study provided a comprehensive analysis of the *Gossypium SOT* gene family and the results might be useful in understanding the role of *SOT* in plant development.

## Results

### Identification, characterization and chromosomal distribution of *SOT* genes in four cotton species

According to the results of BLASTP and HMMER 3.1, a total of 245 *SOT* genes were identified from four cotton species, including 46 genes of *G. arboreum*, 46 genes of *G. raimondii*, 76 genes of *G. barbadense* and 77 genes of *G. hirsutum*. Protein sequence analysis indicated that all *SOT* gene proteins encoded a wide range of amino acids ranging from 60 to 672, with an average molecular weights (Mw) at 32.48 kDa and isoelectric points (pI) at 6.58. Subcellular localization analysis showed that 70.6% of 245 *SOT* genes were localized in the cytoplasm, which may be consistent with their functions as transferases. The *SOT* gene names, locus IDs and other characteristics were listed in Additional file 1: Table S1.

**Fig. 1:**
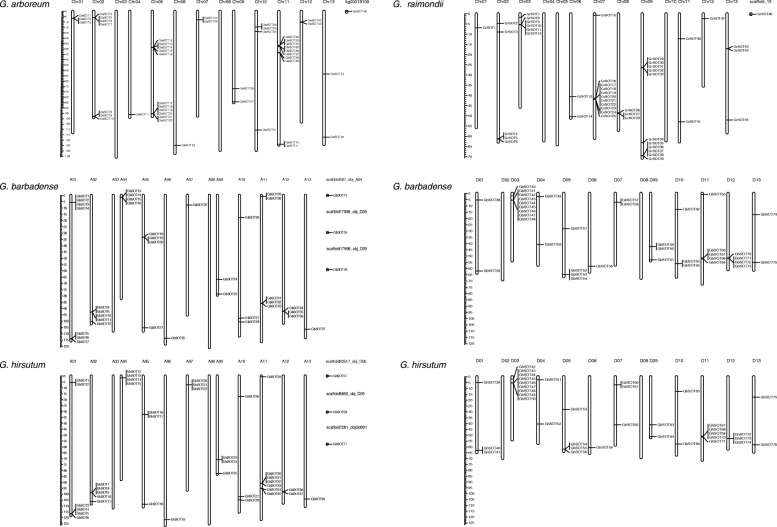
Chromosome distribution of *SOT* genes of four cotton species. The cotton species name was on the left of graphic and chromosome name was at the top of each bar. The vertical scale on the left showed the size of chromosomes and black lines indicated the corresponding position of genes. The scale of the chromosomes was millions of base pairs (Mb). The gene names was correspond to those in Additional file 1: Table S1

245 *SOT* genes distributed unevenly on the chromosomes in four cotton species (Fig. 1). Chr09 of *G. raimondii* contained the largest number of *SOT* genes (11). By contrast, Chr03/ Chr08 of *G. arboreum*, Chr04/ Chr05/ Chr10 of *G. raimondii*, A03/ A08/ D02/ D08 of *G. barbadense* and A03/ A08/ D02/ D08 of *G. hirsutum* contained none of *SOT* genes. In addition, the distribution of *SOT* genes in *G. barbadense* and *G. hirsutum* showed some similarities. So, we further analyzed the collinearity of the *SOT* gene across these four genomes.

### Collinearity and duplication analysis of *SOT* genes

**Fig. 2:**
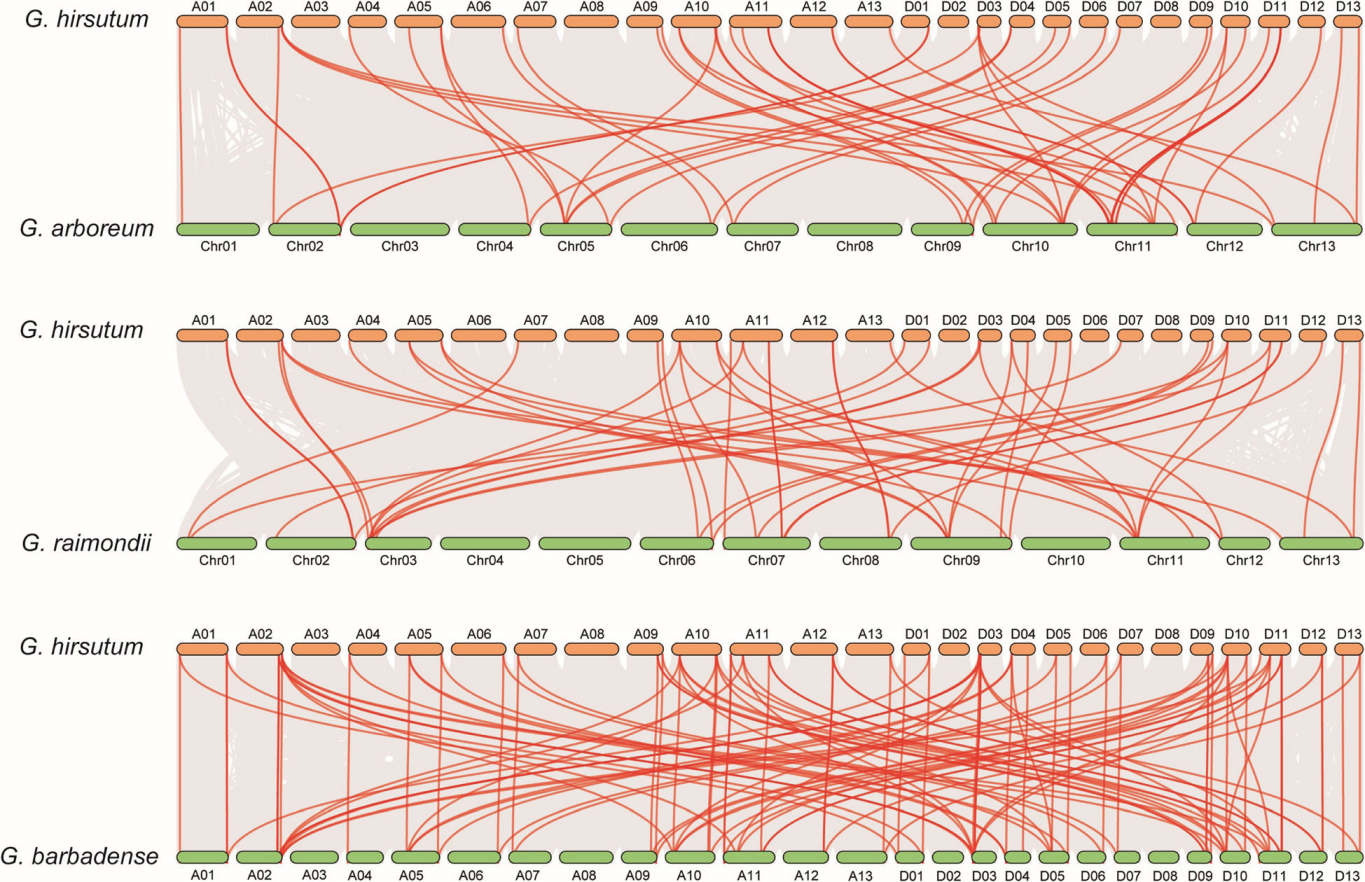
Collinearity analyses of SOT genes among *G. hirsutum*, *G. barbadense*, *G. arboretum* and *G. raimondii*. From top to bottom, three graphics displayed the collinear relationship between *G. hirsutum* and *G. arboretum*, *G. hirsutum* and *G. raimondii*, *G. hirsutum* and *G. barbadense,* respectively. Grey lines in the background showed the collinear relationship across the whole genome, while the red lines predominantly displayed the collinear SOT gene pairs

We found out all the homologous genes among these four cotton genomes to analyze the collinearity relationships of *SOT* genes (Fig. 2 and Additional file 1: Table S2). Among all the 77 *SOT* genes of *G. hirsutum*, 39 *GhSOTs* had intergenomic homologous genes in *G. arboretum*, 37 homologous genes in *G. raimondii* and 49 homologous genes in *G. barbadense*, respectively. In total, we identified 32 pairs of common homologous *SOT* genes in the four cotton species.

**Fig. 3:**
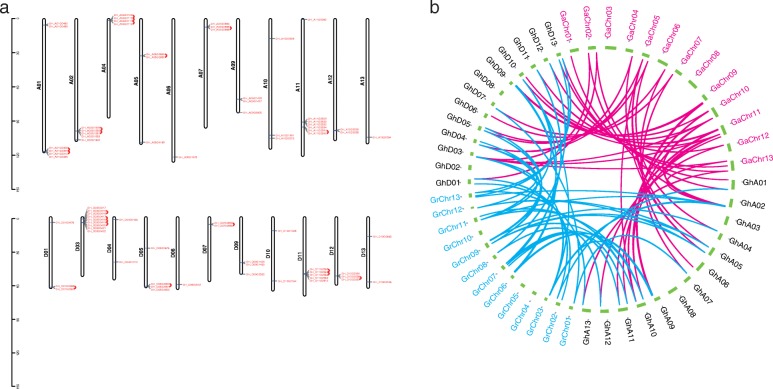
Duplication and synteny of SOT genes among *G. hirsutum*, *G. arboretum* and *G. raimondii*. **a** Localization and duplication of *SOT* genes on *G. hirsutum* chromosomes. Tandem duplication gene pairs were marked with red curve lines. **b** The synteny of SOT genes between *G. hirsutum* and two diploid cottons, *G. arboretum* and *G. raimondii*. Red lines connected the homologous genes between *G. hirsutum* and *G. arboretum*, blue lines connected the homologous genes between *G. hirsutum* and *G. raimondii*, respectively

Previous studies in *Gossypium* showed that gene families always expanded through tandem, whole-genome and segmental duplications [34, 35]. In *G. hirsutum*, 20 pairs of tandem duplication gene pairs (32 genes) distributing on 12 chromosomes were found (Fig. 3a and Additional file 1: Table S3). In addition, 16 gene pairs of replications were categorized as WGD/segmental duplicates. The remaining gene replication mechanisms were detected as proximal or dispersed. As a result, tandem replication might primarily contribute to the expansion of the *SOT* gene family during the evolution of *G. hirsutum*. In order to understand the collinearity of the *SOT* gene family between *G. hirsutum* and two diploid cottons ancestors, we also identified these linked gene pairs (Fig. 3b). 56 collinear gene pairs were identified between *G. hirsutum* and *G. arboretum*, and 29 of them belonged to At subgroup in *G. hirsutum*. 48 collinear gene pairs were also found between *G. hirsutum* and *G. raimondii*, and 22 genes were Dt subgroup in *G. hirsutum*.

### Phylogenetic analysis of *SOT* genes

**Fig. 4:**
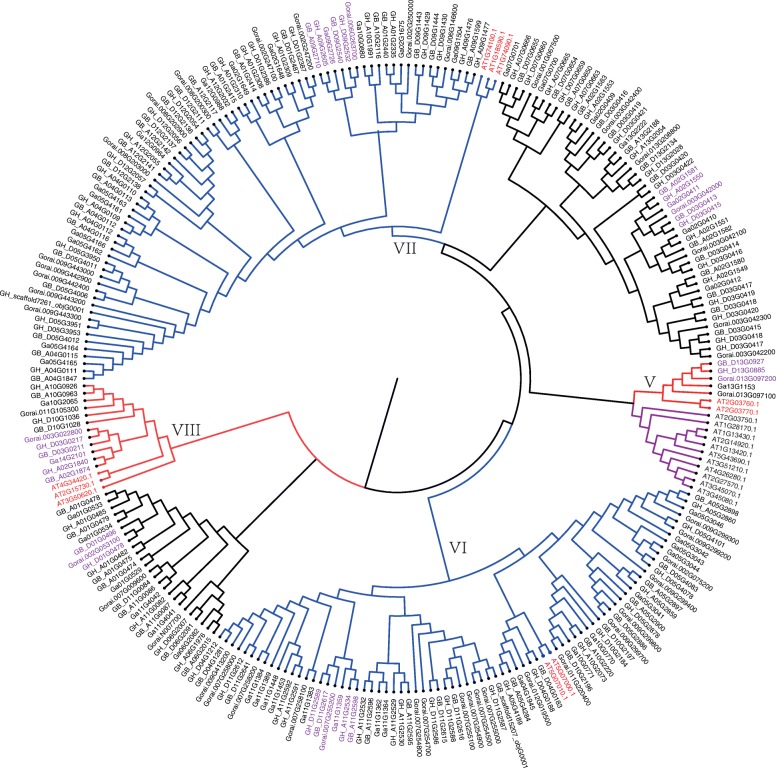
Phylogenetic tree of 266 SOT genes from *Gossypium* and *Arabidopsis*. Neighbor-Joining tree was constructed by Mega X program with the full-length amino acid sequence of the *SOT* genes. The number of subfamily was marked according to the classification results of genes in *Arabidopsis*. Genes marked in red came from *Arabidopsis***.** Genes marked in purple indicated that the clustered genes came from two tetraploids and one diploid

From the phylogenetic tree constructed by all members of the *SOT* genes (Fig. 4), 170 of the 245 *SOT* genes were distributed in 4 subfamilies, and the remaining 75 were separated into two clades. The subfamilies VII and VI were the largest two subfamilies, containing 78 and 75 members, respectively. Subfamily V was the smallest one, including only five genes. The *SOT* genes from four cotton species were more closely related than the genes from *Arabidopsis*. In addition, at the end of the branch, there were many clades where three genes were clustered together. Generally speaking, of the three genes, two genes are from the At subgroup of tetraploids, one from *G. arboretum*; or two genes from the tetraploid Dt subgroup, one gene from *G. raimondii*. This was consistent with the fact that tetraploids came from two diploids [36]. However, after the formation of tetraploids, the relationship between the two tetraploids was closer than that between their ancestors.

### Structural characterizations and conserved motif analyses of *GhSOT* genes

**Fig. 5:**
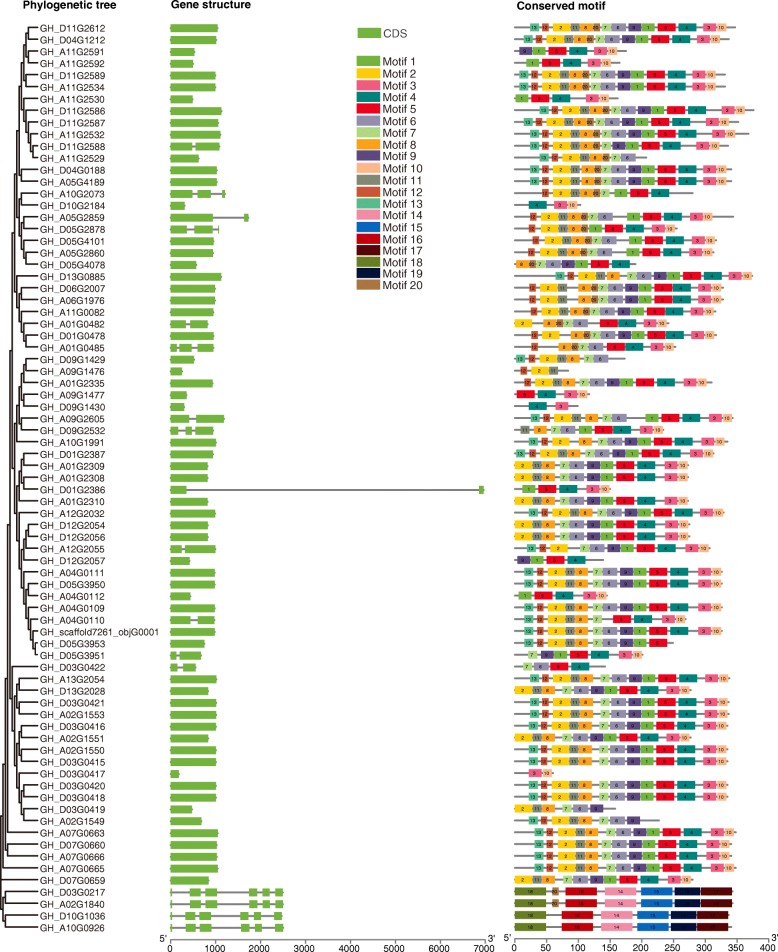
Conserved motif and gene structure of *GhSOT* genes. The phylogenetic tree was generated using protein sequences of 77 *GhSOT* genes. Intron/exon structure of *SOT* genes was analyzed by GSDS. Green boxes standed for exons; grey lines for introns. 20 conserved motifs were identified by MEME. Different color boxes with number represented different motifs

The gene structure of *SOT* genes was analyzed according to the gene annotation files and displayed in Fig. 5. Results showed that the exon numbers ranged from 1 to 6, with an average of 1.5. The great majority of genes contained less than 3 exons, and most contained only one exon. Classically, genes in the same evolutionary branch had similar structures, which shared a conserved gene structure pattern in terms of intron/exon number and intron/ exon length.

20 conserved motifs of *GhSOT* genes were identified through the MEME program (Fig. 5 and Additional file 1: Table S4), with a width ranged from 11 to 50 amino acids. The number of conserved motifs in different genes varied from 2 to 14, however, in the same branch of the phylogenetic tree, the number and type of conserved motifs were similar. Motif 4 appeared in 66 genes and was common to almost all *GhSOT* genes, followed by Motif 5, 3, 10, 7, 1 (appearing in more than 60 genes). The gene structures and conserved motifs of the four genes on the same evolutionary clade, *GH_D03G0217*, *GH_A02G1840*, *GH_D10G1036* and *GH_A10G0926*, were different from other genes, which may lead to changes in evolutionary speed and function.

### RNA-Seq expression profile of *GhSOT* genes

**Fig. 6:**
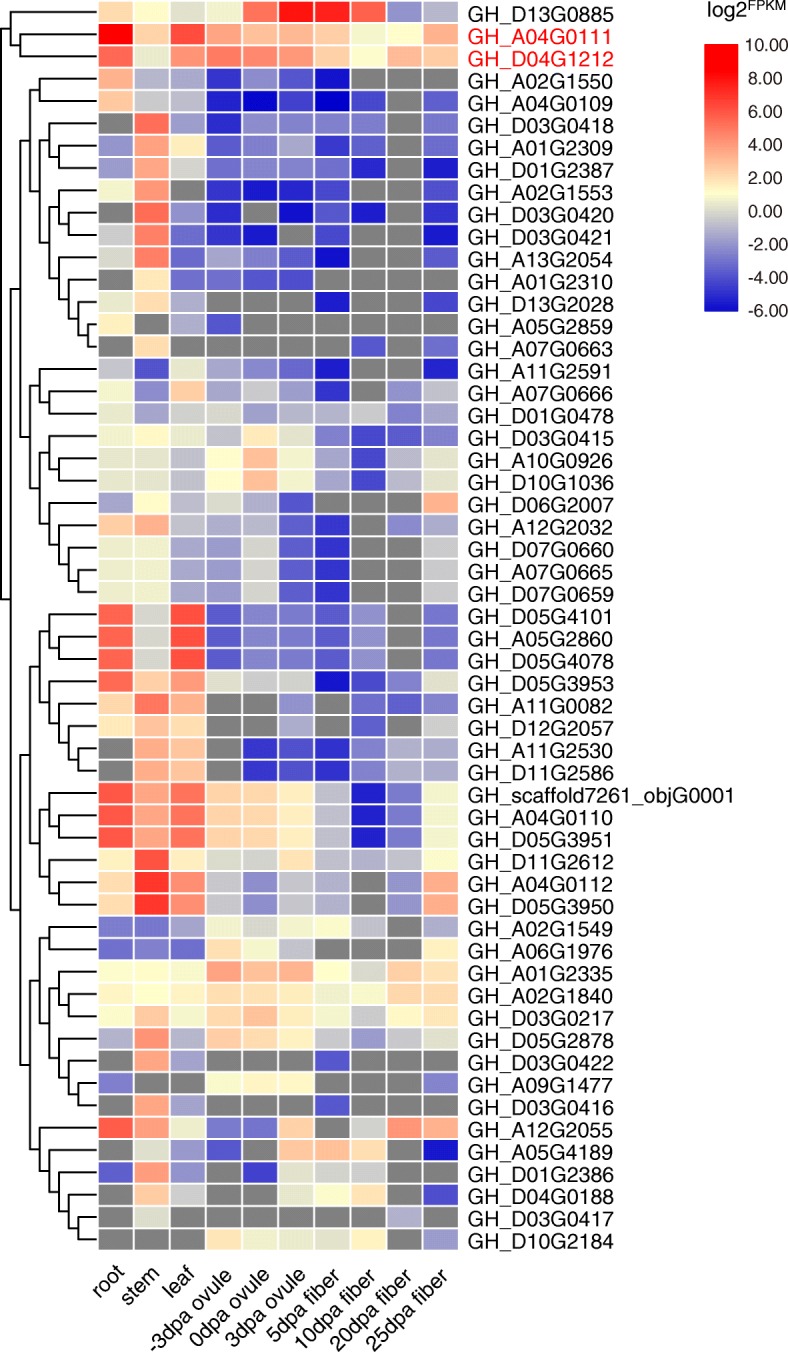
Transcriptome expression of *GhSOT* genes in different tissues and developmental stages (FPKM> 1). Expression levels were illustrated by graded color scale, red indicated high FPKM value, blue indicated low abundance, while grey indicated none expression. Genes marked in red represented constitutively and highly expression in 10 tissues

Firstly, 21 *GhSOT* genes with expression levels less than 1 at 10 different stages were eliminated. The raw data of the remaining 56 *GhSOT* genes were normalized to log2^FPKM^ and the heatmap of the expression was shown in Fig. 6. Most genes exhibited characteristics that were specifically expressed during the different stages. 16 genes were constitutively expressed in 10 tissues, especially the expression values of *GH_A04G0111* and *GH_D04G1212* were more than 1 at all stages. Most of the *GhSOT* genes exhibited a high level of expression in the stem, leaf, and the initial stage of fiber development (− 3, 0, 3 dpa ovule). This indicated that *SOT* genes might be associated with fiber development in cotton. As reported in previous study [37], a lot of loci related with fiber quality were clustered on chromosomes D11. In this study, there were two *SOT* genes located on chromosomes D11, and one of them was specifically expressed in several tissues. So, we further performed experiments to understand the characteristics and functions of *GhSOT67* (*GH_D11G2586*).

### Tissue and subcellular localization analysis of *GhSOT67*

**Fig. 7:**
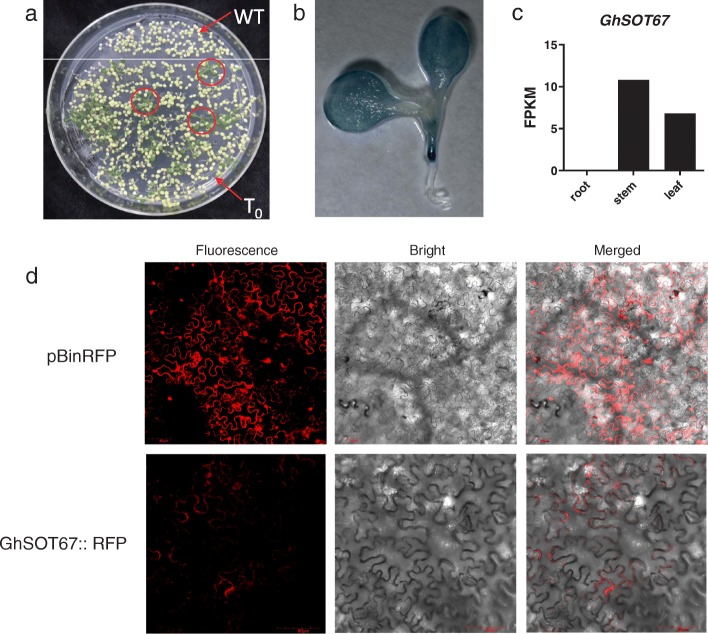
Tissue and subcellular localization of *GhSOT67*. **a** Transformants were planted on half-strength MS medium containing 50 μg/mL kanamycin, the red circle showed the positive plants. **b** GUS staining analysis of the positive transgenic lines. **c** The FPKM value of *GhSOT67* according to the transcriptome data. **d** Subcellular localization of RFP fusion proteins of *GhSOT67* in infected tobacco leaves

To investigate the tissue localization of *GhSOT67*, a recombinant vector of pGhSOT67::GUS was constructed and transformed into *Arabidopsis* mediated by *Agrobacterium tumefaciens* cells (GV3101). Multiple positive transformants were screened, soaked in the GUS staining solution and the most typical one was shown in Fig. 7a and b. The results showed that the staining in blue color was found in the stem and leaf of the transformant plant, which was consistent with the expression of the transcriptome expression of *GhSOT67* (Fig. 7c). This expression pattern had also been reported in *Arabidopsis* [8].

According to the online tool CELLO, *GhSOT67* was predicted to be localized in the cytoplasm (Additional file 1: Table S1). To verify this, full-length CDS of *GhSOT67* without initial condon was ligated with pBinRFP vector. The control empty vector pBinRFP was present all over the cell, including the nucleus, membrane and cytoplasm (Fig. 7d). By contrast, the GhSOT67::RFP fusion protein was mainly localized in cytoplasm, confirming the previously predicted result.

### Virus-induced gene silencing (VIGS) of *GhSOT67* in cotton

In order to investigate the relationship between *GhSOT67* gene and fiber development, we performed VIGS on a cotton variety, J02. The empty vector pYL156 was used as a negative control. The recombinant vector pYL156:CLA1 could induce a leaf bleaching phenotype, therefore, it was served as a positive control to indicate the success of gene silencing.

**Fig. 8:**
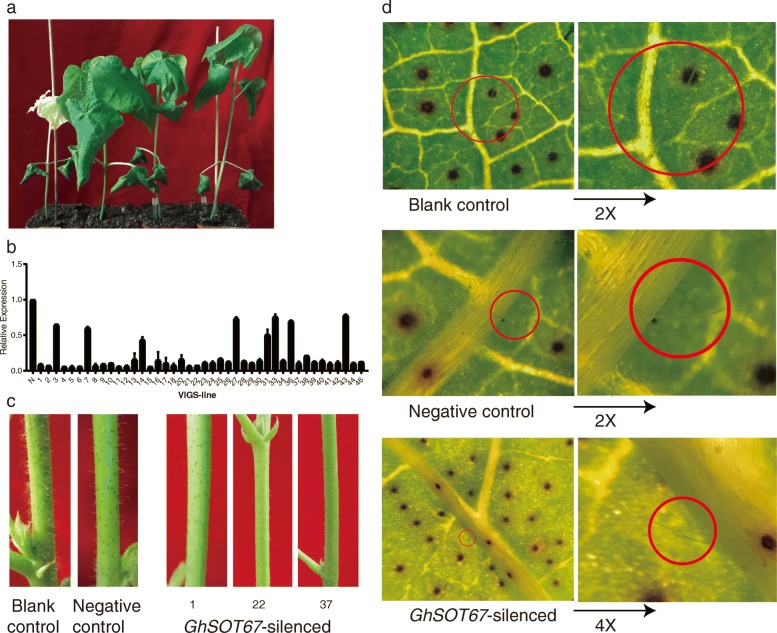
*Agrobacterium*-mediated VIGS of *GhSOT67* in cotton. **a** Phenotypes of gene silencing plants. The plants from left to right were *CLA1*-silenced, blank control, negative control and *GhSOT67*-silenced. **b** The expression levels of *GhSOT67* in the negative control and silenced cotton plants conducted through qRT-PCR. N referred to the negtive control (Agrobacterial culture suspension of pYL156 only). **c** The stem hair of cotton after the induction. Left: blank control; Middle: negative control; Right: three lines of *GhSOT67*-silenced plant. **d** The leaf hairs of cotton observed under an optical microscopy after the induction. Upper: blank control under 10-fold visual field, the right one was the twice magnified leaf hair; Middle: negative control under 15-fold visual field, the right one was the double enlarged leaf hair; Lower: *GhSOT67*-silenced plant under 15-fold visual field, the right one was the fourfold enlarged leaf hair

17 days after the induction, the albino phenotype occurred on the positive control plants (Fig. 8a), proving that VIGS was successful. The expression of *GhSOT67* after gene silencing was firstly verified by PCR compared with *Histon3*. Subsequently, the results of qRT-PCR revealed that the level of gene expression of most *GhSOT67* silenced plants decreased by more than 80% (Fig. 8b). As shown in Fig. 8c, after 1 month of the treatment, the number of stem hairs in *GhSOT67* silenced plant decreased evidently, comparing with the blank and negative control plants. In the meantime, the length of stem and leaf hairs of *GhSOT67* silenced plants was obviously shorter than that of control plants (Fig. 8c and d). The stem and leaf hairs, as well as cotton seed fiber, were originated from the single cell layer, which might have similar fiber differentiation and development mechanisms [38–40]. Accordingly, the results suggested that *GhSOT67* might be involved in the fiber development process.

## Discussion

In recent years, the nuclear genome sequences of *G. arboreum*, *G. raimondii*, *G. hirsutum*, *G. barbadense* and *G. hirsutum* have been published successively [41–44], further deepening the understanding of cotton genomics and genetics, which provides a possibility for exploring *SOT* gene family members and their phylogenetic relationships. Here, we identified a total of 245 *SOT* genes from four cotton species, according to the sequence identity of proteins. The number of *GhSOT* and *GbSOT* genes were more than that of *SOT* genes in two diploid cotton, possibly due to the polyploidization event occurred in two tetraploid cotton about 1.5 million years ago (Mya) [36].

Gene duplication is considered to be the main driver of evolution, leading to functional differentiation and diversification [45]. Gene duplication mainly includes three forms such as tandem, whole-genome and segmental duplications. In this study, we found that tandem replication might primarily contribute to the expansion of the *GhSOT* gene family, as well as several other replication methods exist. On the bases of the previous reports in *Arabidopsis*, *SOTs* had been divided into 8 groups [1, 9]. Phylogenetic analysis demonstrated that 245 *SOT* genes from Gossypium were cluster with *SOTs* from *Arabidopsis* into 4 clades, except for 75 *SOTs* from *Gossypium*. The convergence of three genes at the end of the evolutionary branch was consistent with previous studies that two diploids were the ancestors of tetraploids [36]. The difference in the number of exons and conserved motifs between genes indicated that the gain and loss of exons may lead to the functional diversity of *SOT* genes closely related to the evolution of *SOT* gene family.

To date, only a few *Arabidopsis SOTs* were functionally characterized. *At5g07000* from group VI was proved to catalyze the sulfation of 12-hydroxyjasmonates, thus causing inactivation of jasmonic acid in plants [16]. For another *Arabidopsis SOT*, *At3g45070* from group II, had been found to specifically bind to flavonols [1]. For the *GhSOT* gene members, we paid particular attention to those that might play crucial roles in plant growth or fiber development. Combining the transcriptome expression of *GhSOT* genes with the fiber-quality-related loci reported previously [37], *GhSOT67* was selected to further understand its characteristics and functions. For the localization analysis, *GhSOT67* was estimated to express in cytoplasm and locate in stem and leaf tissue. These features would be related to its function as a catalyst [8]. Transcriptome expression showed that *GhSOT67* was specifically expressed in several tissues and the initial stage of fiber development (− 3, 0, 3 dpa ovule). In addition, *GhSOT67*-silenced plants treated by VIGS showed a shorter length of stem and leaf hairs than that of control plants. According to the results of phylogenetic cluster, *GhSOT67* belonged to group VI, it might have similar function to *At5g07000* that can catalyze the inactivation of jasmonic acid. So we speculated that when *GhSOT67* was silenced, jasmonic acid could not be sulfated and accumulated in the plant, then the length of stem and leaf hairs was shortened. Taken together, these results suggest that *GhSOT67* may involve in cotton fiber development. However, the detailed correlation between *SOTs*, jasmonic acid and fiber development remains to be further verified.

## Conclusion

In this study, a comprehensive analysis including chromosomal location, collinearity and duplication, gene structure and expression patterns of the *SOT* gene family in *Gossypium* was first performed. To summarise, we isolated a total of 245 *SOT* genes in the genome of *G. arboreum*, *G. raimondii*, *G. barbadense* and *G. hirsutum,* and further classified the *SOT* genes into four groups based on the orthologous relationships comparing with *Arabidopsis*. Tandem replication primarily contributed to the expansion of *SOT* gene family in *G. hirsutum*. Expression profiles of the *GhSOTs* in various tissue and developmental stages implied that *GhSOTs* might be involved in the fiber development. In addition, gene silencing by VIGS significantly induced the expression of *GhSOT67* and shortened the length of stem and leaf hairs. Taken together, these findings indicated that *SOT* genes might be associated with fiber development in cotton.

## Methods

### Database search and sequence retrieval

The genome files and protein sequences of two diploid cottons [41, 46] (*G. arboreum* L., *G. raimondii* Ulbr.) and two tetraploid cottons [44] (*G. hirsutum* L., *G. barbadense* L.) were downloaded from the Cotton Functional Genomics Database (CottonFGD) (https://cottonfgd.org/) [47]. The protein sequences of *Arabidopsis thaliana* (L.) were obtained from the *Arabidopsis* Information Resource (TAIR) (https://www.arabidopsis.org/). Based on the sequence similarity of the translated products, the *Arabidopsis* whole genome contains 21 genes encoding the *SOT* protein (*AtSOT*) [1] and all 21 *Arabidopsis SOT* proteins were extracted using TBtools (https://github.com/CJ-Chen/TBtools/releases) [48].

Two methods were used to search *SOT* genes in four cotton species. Firstly, 21 *Arabidopsis SOT* proteins were used as query sequences against the four cotton protein sequences files with default parameters (e-value <1e-5) through BLAST algorithm for Proteins (BLASTP) search. The candidate *SOT* genes of each cotton species were named separately, such as *GhSOT* from *G. hirsutum* and *GbSOT* from *G. barbadens*. Secondly, the hidden Markov model seed file (Stockholm format) of sulfotransferase domain (PF00685) were acquired from Pfam (http://pfam.xfam.org/) and used as a query sequence searching for candidate *SOT* protein sequences against the four cotton protein sequences files by Hmmer 3.0 (http://hmmer.org/), with default parameters. The *SOT* protein sequences with e-value less than 15 were preserved. Then, we merged all hits obtained above and discarded the repetitive sequences. All non-redundant protein sequences were further checked the conserved domains of the protein using the NCBI Conserved Domain Database (https://www.ncbi.nlm.nih.gov/cdd) in automatic mode (threshold = 0.01, maximum hits =500).

Finally, the candidate *SOT* genes were further manually confirmed to eliminate the pseudo sequences and the position in the cell was predicted according to the online tool CELLO v2.5 (http://cello.life.nctu.edu.tw/) [49]. The molecular weight (Mw) and isoelectric points (pI) of the candidate *SOT* genes were predicted using the online ExPASy server (http://web.expasy.org/compute_pi/) [50].

### Chromosomal mapping and phylogenetic analysis

Chromosomal position and gene structure information of *SOT* genes were obtained from four cotton gene annotation files, and these *SOT* genes were mapped separately on the corresponding chromosomes using the MapChart software (https://www.wur.nl/en/show/Mapchart/).

The full-length amino acid sequence of the *SOT* genes from both *Arabidopsis* and *Gossypium* were saved as a fasta format file and used to perform multiple sequence alignments using the ClustalW program with the default settings. Subsequently, we constructed the neighbor-joining (NJ) tree in MEGA X, the parameters were set as follows: 1000 bootstrap replicates, Jones-Taylor-Thornton (JTT) substitution model, and partial gap deletion mode with a cut-off value of 80%.

### Intron/exon distribution and conserved motif analysis

The gene structure of *SOT* genes was analyzed using Gene Structure Display Server 2.0 (GSDS, http://gsds.cbi.pku.edu.cn/) [51]. The conserved domain motifs of the *SOTs* were determined by Multiple Em for Motif Elicitation (MEME) (http://meme-suite.org/tools/meme) [52] according to the following parameters: site distribution was set at 0 or 1 occurrence per sequence, the width of motifs ranged from 6 to 50, the maximum number of motifs was 20. All the characteristic results of *SOT* genes were visualized and integrated into graphics by Tbtools.

### Gene expression analysis

The fragments per kilobase of exon per million fragments mapped (FPKM) values were acquired from the transcriptome data of *G. hirsutum* cv. TM-1 [53]. The expression values of three different tissues and seven different stages of fiber development, − 3 dpa (day post anthesis) ovule, 0 dpa ovule, 3 dpa ovule as well as 5, 10, 20, and 25 dpa fibers, were considered and the genes with FPKM values more than 1 at least one stage were further analyzed. The expression of the *SOT* gene was estimated to be normalized in the form of log2^FPKM^ and displayed in the heat map.

### Plant materials

A cotton variety, J02, was provided by Germplasm Repository of Institute of Cotton Research, Chinese Academy of Agricultural Sciences (CRI of CAAS, Anyang, Henan province, China) only for scientific research purpose. J02 was sown in mixed soil (vermiculite:humus = 1:1) and cultured in an incubator with a 16 h /8 h (light/ dark) photoperiod at 28 °C and 25 °C respectively till the cotyledons were fully unfolded.

*Arabidopsis thaliana* ecotype Colombia (Col-0) and tobacco (*Nicotiana benthamiana*) were also provided by CRI of CAAS and grown as recipient materials in the following ways. The seeds were grown on agar-solidified Murashige and Skoog (MS) medium by dropper, and after 48 h of hypothermia, the culture dishes were placed in an incubator with a 16 h / 8 h (light / dark) photoperiod at 24 °C and 22 °C respectively. When the cotyledons were unfolded, the seedlings were transplanted into sterile mixed soil (vermiculite:humus = 1:1).

### Construction of target gene vectors and their inoculation treatment

In order to perform the tissue location of *GhSOT67*, 1500 bp promoter sequence upstream of the gene was amplified and inserted into the two restriction sites (HindIII and BamHI) of pBI121 vector. The *Agrobacterium tumefaciens* cells (GV3101) containing constructed vector was transformed into *Arabidopsis* plants according to the floral dip method [54]. The wildtype and transgenic plants were grown under conditions mentioned above. Positive transformants were screened by planting on half-strength MS medium containing 50 μg/mL kanamycin and confirmed by PCR and β-glucuronidase (GUS) staining.

The CDS of *GhSOT67* without initial codon was inserted into the SalIrestriction site of the pBinRFP vector [55] to construct the translational RFP fusion constructs. The recombinant plasmid was transformed into *Agrobacterium tumefaciens* strain LBA4404 and inoculated into the second or third leaves on top of the tobacco according to the protocols [56]. The vector of pBinRFP (RFP alone) was also transformed into the tobacco leaves which was planted at the same time and in the same condition as the control. Finally, the infected tobacco leaves were wrapped in tinfoil, placed in a dark environment for 24–48 h and observed under an optical microscopy with CCD camera (Leica Microsystems, Germany) [57].

For the virus-induced gene silencing (VIGS) experiment, an specific 300-bp sequence selected from the *GhSOT67* was amplified with two restriction sites at both ends (SpeI and AscI). Firstly, the PCR amplification product was cloned into pMD19 T vector. Both the resultant construct and pYL156 were digested with SpeI and AscI, and connected through ligation buffer solutionI to form pYL156:GhSOT67. The plasmid was transformed into *Agrobacterium tumefaciens* LBA4404 for infecting cotton. Agrobacterial culture suspension of pYL192 was respectively mixed with others equally as an auxiliary carrier. Agrobacterial culture suspension of pYL156 (negtive control), pYL156:CLA1 (positive control) and pYL156:GhSOT were separately injected into fully expanded cotyledons of cotton variety, J02, before the true leaves hadn’t yet emerged. Ten strains of J02 were reserved for wild type (blank control), 10 strains were injected with pYL156 and pYL156:CLA1 respectively, and 45 strains were injected with pYL156:GhSOT67. Experimental procedures and methods of operation were used as described by ref. [58].

### Collections, RNA isolation and qRT-PCR analysis

About 2 weeks post infiltration, when true leaves appeared albino phenotype, the leaves of the J02 were put into the liquid nitrogen immediately and stored at − 80 °C for RNA isolation and analysis. Total RNA was extracted via the RNA extraction kit (TIANGEN, Beijing, China). First-strand cDNA was synthesized using PrimeScript™ RT reagent Kit with gDNA Eraser (TaKaRa, Japan). The quantitative real-time (qRT)-PCR analysis was completed on 7500 Fast Real-Time PCR system (Applied Biosystems, Inc., California USA) with SYBR Premix Ex Taq (TaKaRa, Japan). The *Histon3* gene were used as an endogenous control to normalize gene expression. The relative expression levels of *GhSOT67* gene after infiltration was calculated using the 2^-ΔΔC^_T_ method [59].

All the gene-specific primers used for amplifications or vector constructions were listed in Additional file 1: Table S5.

## Supplementary information

**Additional file 1: Table S1.** List of *SOT* genes identified in *Gossypium* and their sequence properties. **Table S2.** Duplicated *SOT* gene pairs among four cotton species. **Table S3.** Duplicated *SOT* gene pairs in *G. hirsutum*. **Table S4.** Informations of motifs in *SOT* genes. **Table S5.** Gene-specific primers used for amplifications or vector constructions.

## Data Availability

The datasets supporting the conclusions of the present study are included within this article (and its additional files). The authors are pleased to share any raw data upon request.

## Abbreviations

APS: Adenosine-5′-phosphosulfate
BLASTP: BLAST algorithm for proteins
CDS: Coding sequence
Chr: Chromosome
CottonFGD: Cotton functional genomics database
dpa: Days post anthesis
FPKM: Fragments per kilobase of exon per million fragments mapped
GSDS: Gene structure display server
GUS: β-glucuronidase
JTT: Jones-Taylor-Thornton
Mb: millions of base pairs
MEME: Multiple em for motif elicitation
MS: Murashige and Skoog
Mw: Molecular weights
Mya: Million years ago
NJ: neighbor-joining
PAPS: 3′-phosphoadenosine-5′-phosphosulfate
pI: Isoelectric points
qRT-PCR: Quantitative real-time PCR
RNA-seq: RNA sequencing
SOTs: Sulfotransferases;
TAIR: The *Arabidopsis* information resource
VIGS: Virus-induced gene silencing
WGD: Whole genome duplication
